# Time-restricted feeding in rodent obesity models: impact on body weights, lipid profile and glucoregulation

**DOI:** 10.1038/s41366-025-01948-6

**Published:** 2025-12-19

**Authors:** Joyce Argaistieng, Bavani Visha Doraisamy, Hasseri Halim, Sharifah Sakinah Syed Alwi, Aida Azlina Ali, Sandra Maniam

**Affiliations:** 1 Department of Human Anatomy, Faculty of Medicine and Health Sciences, Universiti Putra Malaysia, Serdang, Selangor Malaysia; 2 Department of Pharmacology & Life Sciences, Faculty of Pharmacy, Universiti Teknologi MARA (UiTM), Selangor, Malaysia; 3 Integrative Pharmacogenomics Institute (iPromise), Universiti Teknologi MARA, Selangor, Puncak Alam Malaysia; 4 Department of Biomedical Science, Faculty of Medicine and Health Sciences, Universiti Putra Malaysia, Serdang, Selangor Malaysia

**Keywords:** Nutrition therapy, Obesity

## Abstract

**Introduction:**

Dietary techniques such as time-restricted feeding (TRF) have received support in recent years due to their ability to improve metabolic health and prevent serious diseases. In scientific research, animal models are widely utilized to understand the physiological impacts of fasting and other dietary interventions, as they have similar physiology to humans. Several feeding windows ranging from 4 to 12 h have been reported in the literature. This review evaluates TRF protocols to determine the most effective feeding window for improving metabolic profiles.

**Methods:**

Several search keywords were utilized and only research articles published within the last fifteen years (2009–2024) were selected. Twelve studies were included in the final analysis to improve transparency.

**Results:**

Obesity was successfully induced within 6 weeks for 100% weight gain in C57BL/6 mice. The shortest duration of TRF intervention in mice is 6 weeks with 10 h of feeding. Meanwhile, induced obesity with 300% weight gain in Sprague-Dawley rats within 12 weeks. The shortest duration of TRF is 6 weeks with 8 h of feeding.

**Conclusion:**

TRF was consistently associated with reductions in body weight and total cholesterol, concomitant with an increase in glucose tolerance and insulin sensitivity in studies where these parameters were assessed. The most effective identified TRF regimen is a 10-h feeding window over 8 weeks in C57BL/6 mice. Future research on obesity may take into account the inclusion of different metabolic challenges to assess if the advantages of TRF are exclusive to any of the challenges or multiple challenges that contribute obesity.

**Limitations:**

A key limitation of this review is the heterogeneity in study protocols. The included studies varied in the duration of feeding hours (ranging from 4 to 12 h) using different rodent models.

This research was funded by the Ministry of Higher Education Malaysia, under the Fundamental Research Grant Scheme (FRGS/1/2023/SKK06/UPM/02/2).

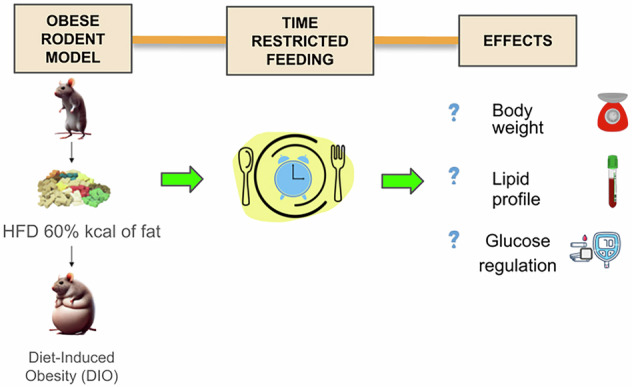

## Introduction

Fasting is commonly referred to as voluntary abstaining from any drinks or food for specific duration of time [1]. The postabsorptive state also known as fasting refers to the duration of roughly 6–12 h after a meal until the next meal consumption [2]. Fasting consists of three stages which are post-absorptive, fasting and starvation. The post-absorptive state is the condition where no glucose or nutrients are taken in. Glycogenolysis which is the process of breaking down glycogen in the liver will provide glucose to both blood and tissues. Once there is no food intake for 18–24 h, the hepatic glycogen is diminished. Gluconeogenesis or also known as the production of glucose from non-carbohydrates sources like fat, lactate and amino acid will occur. Hence, this leads to the de novo production of glucose which is required for energy production [3]. During the 36–48 h fasting state, the insulin levels are lower due to gluconeogenesis. Fuel is produced by fatty acids and ketones as a result of a metabolic change. This also causes lipolysis to rise, which raises the amount of free fatty acids produced [4]. A lower level of insulin is present in the final state which is starvation that sets in after more than 48 h of fasting. The muscle’s ability to oxidize glucose and break down branched-chain amino acids (BCAAs) is suppressed. Gluconeogenesis is limited to the kidneys. The energy comes from the free fatty acids that are liberated during lipolysis activity [4]. Fasting has been often associated with a lot of health benefits such as reducing the body weight, reducing the blood sugar level, improving insulin level and blood pressure [5]. Consequently, over the last decade, varieties of intermittent fasting and time-restricted feeding have evolved [3].

Intermittent fasting (IF) is a practice of restraining from consuming any food or drinks that will last for 24 h by alternating days or 16 h everyday [6]. In addition, IF can also be practiced by having the last meal on a certain day at 8.00 pm and begin fasting after waking up on the next day. The fasting will continue until the afternoon of that day. A sum of 16 h of fasting is gained by totaling the sleeping hours with morning fasting hours [3]. IF leads to a decreased level of blood glucose and insulin which creates an improvement in glucagon secretion to refine glycogenolysis [6]. There are many types of IF such as alternate-day fasting (ADF), fast-mimicking diet (FMD), religious fasting and TRF [7].

Commonly, ADF is practiced with one day of eating, followed by one day of fasting or five days of eating with 2 days of fasting (5:2 diet) [3]. This technique permits usage of 20–25% of required energy during fasting [7]. FMD is a plant-based constituent that has reduced levels of protein and sugar but increased levels of fat [8]. A vegetarian diet does not necessarily limit calories and concentrates only on plant-based nutrition without precise macronutrient limits, but the FMD is low in calories over a short period of time and emphasizes certain macronutrients to produce a similar effect as fasting. FMD is often observed as a fruitful solution for the majority of people to gain a similar result as in fasting due to its nutritional adjustment advantageous for cardiometabolic disease and to control weight [3].

Religious fasting is executed to fulfil certain spiritual requirements and is identified as a type of nutritional model that differs in terms of caloric restriction and food intake [9]. Ramadan is a religious month of fasting for 29–30 days which is well-known among Muslims. The fasting starts from sunrise and ends at sunset without any food or drink consumption [3].

In addition to protocols involving prolonged daily fasting, there are time-sensitive IF approaches in which food intake is restricted to specific periods. TRF has emerged as a practical and effective IF regimen with significant potential for improving metabolic health. TRF is an innovative dietary strategy that reduces the daily eating duration without changing the total caloric consumed [10]. The TRF concept is designed to reduce the daily eating duration without requiring a reduction in the total caloric intake. Contrarily, there are some studies that have reported reduction in the caloric consumption in animal (or human) studies involving TRF protocols [11, 12]. As TRF limits the feeding duration, it naturally reduces the calorie intake. It can be concluded that TRF focuses on when food is consumed but depending on the study as the total calories consumed may or may not vary. To date, most of the studies on TRF range from 4–12 h of feeding duration. The TRF procedure is very pleasing as it allows individuals to adhere to their eating preference to improve consistency with the diet protocol. Research has proven that TRF provides benefits without caloric restriction [13–15]. For instance, a randomized isocaloric study on TRF revealed a decrease in blood sugar levels leading to better insulin resistance [11]. Moreover, TRF helps reduce body weight gain, improves glucose tolerance and gut function and reduces atherogenic lipid levels [10].

To understand the relationship between obesity and insulin resistance, mice model is commonly utilized. HFD induces obesity and insulin resistance in the mice model within a short period of time [16]. Hence, by *ad libitum* feeding the mice with HFD of 42–60% of kcal of fat, it leads to an induction of obesity [16]. There are specific diets used to induce obesity in rodents and diet-induced obesity (DIO) models are the most common ones. In this model, the diets differ with the fat composition of 10%, 45% and 60% [17]. A diet with approximately 10% of fat is identified to be normal for rodents. Subsequently, in studies involving the DIO model, the control group will receive a fat diet of 10% while the experimental group will receive 45% or 60% of HFD [17].

Both mice and rats are the most commonly used laboratory animals. Mice are often utilized as an animal model due to various factors such as small size and short life cycle. Besides, mice are used to mimic the diseases in humans to identify the potential treatments [18]. For instance, diabetes, obesity and Parkinson’s disease. The C57BL/6 J mouse is a well-established model for studying human obesity-related metabolic disorders owing to its high susceptibility to DIO and insulin resistance when fed a HFD [19]. This strain also exhibits a predisposition to metabolic dysfunction which is associated with glucose tolerant and gut microbiota alteration [20].

On the other hand, rats have several criteria making them a more suitable animal model. Just like mice, rats are small in size, short life cycle but they are very easy to handle in a laboratory. Rats are commonly used in research related to nutrition, neurology and genetics [18].

## Methodology

### Search strategy

A search was conducted manually using the Scopus and PubMed database from 2nd February to 6th March in 2024 and only peer-reviewed, published articles available up to March 2024 were included. The combination of descriptors used was “time-restricted feeding”, “diet-induced obesity”, “animal model” and “in vivo”. Each publication was independently screened across multiple databases. The articles that were published within 15 years (2009–2024) written in English language or English translation provided for full-free text focusing only on mice and rats were selected. The search was restricted to the past 15 years to ensure that the evidence included reflects recent methodologies, clinical practices, and scientific relevance of TRF. Non-English studies without translations were excluded to maintain methodological consistency and to minimize the risk of misinterpretation that could arise from language barriers. A formal quality scoring using SYRCLE’s risk of bias tool for animal studies was applied as shown in Table 1.

**Table 1:** A formal quality scoring using SYRCLE’s risk of bias tool for animal studies was applied.

Domain	Question	Example in TRF study	Risk rating
1. Sequence generation	Was the allocation of animals to groups random?	The mice were randomly assigned to groups using a random number table.	Low risk
2. Baseline characteristics	Were groups similar at baseline?	Groups had similar average body weight before intervention.	Low risk
3. Allocation concealment	Was group allocation concealed?	The method of allocation concealment was not reported.	Unclear risk
4. Random housing	Were animals housed randomly during the experiment?	Housing not mentioned.	Unclear risk
5. Blinding of caregivers/investigators	Were those administering interventions blinded?	No blinding mentioned.	High risk
6. Random outcome assessment	Were the animals housed randomly during the experiment?	The order of outcome assessments was not reported.	Unclear risk
7. Blinding of outcome assessment	Were assessors blinded?	Body weight measured by lab staff who knew group assignments.	High risk
8. Incomplete outcome data	Were all animals included in the analysis?	2 mice were excluded due to illness and the reasons were reported.	Low risk
9. Selective outcome reporting	Were all outcomes reported?	A study protocol was not available but reported outcomes appeared complete.	Unclear risk
10. Other bias	Any other sources of bias?	The sample size calculation was not reported.	High risk

Interpretation

1 **Strengths**: Random allocation, balanced baseline, complete data

2 **Weaknesses**: No blinding, poor reporting on housing, no sample size justification.

3 **Overall**: Mixed quality, with potential risk of bias.

4 Low risk = Methods are clearly described, appropriate and unlikely to introduce bias.

5 High risk = Clear methodological problems that could bias results.

6 Unclear risk = Not enough information is provided to judge.

### Eligibility criteria and study selection

The articles were thoroughly reviewed by reading the title and abstract for each of the articles to evaluate the eligibility. Articles without full access or full text were excluded while the articles that emphasize on the intervention of TRF on rodents with obesity were included.

The population, intervention, comparison, and outcome (PICO) is a concept used by researchers to design research questions and refine the quality of literature [21]. The PICO is referred to as an inclusion criteria. The population comprises mice and rats that are fed with HFD to induce obesity or also known as DIO model. This review analyzes TRF intervention conducted at different durations (4, 8, 9, 10, 12 h). The DIO model will be compared with other study designs of TRF conducted at different durations. The outcome comprises blood glucose level, insulin level, lipid profile and body weight gain after TRF intervention.

Studies on diets that focus on other types of IF such as (ADF, periodic fasting, Ramadan fasting, 5:2 diet), animals other than mice and rats, surgically-induced obesity, studies without the induction of obesity, human and in vitro, studies with an intervention before obesity induction or using pregnant or menopause mice/rat are not included in this review. The summary of the inclusion and exclusion criteria as shown in Table 2. A table summarizing the PICO elements for each included study as shown in Table 3.

**Table 2:** Inclusion and exclusion criteria used to evaluate the studies.

Inclusion	Exclusion
Diet-induced obesity	Drug-induced obesity
Any mice/rat strain	Surgically-induced obesity
Original articles	Studies without induction of obesity
Studies that focus on induction and evaluation of obesity	Studies with cell culture
Published within the last 15 years	Studies with humans
Articles in English or that had English translations available	Studies with other animals than mice/rat
In vivo studies	In vitro studies
	Studies with an intervention before obesity is induced
	Studies using pregnant or menopause mice/rat
	Studies that include other types of IF except TRF

**Table 3:** Summarizing the PICO elements for each included study.

Study	Population	Intervention	Comparison	Outcomes
Sherman et al. [22]	C57BL/6 mice fed with HFD of 42% kcal fat	TRF (18 weeks)	HFD ad libitum	Blood glucose, insulin, lipid profile, body weight
Hatori et al. [27]	C57BL/6 J mice fed with HFD of 61% kcal fat	TRF (18 weeks)	HFD ad libitum	Blood glucose, insulin, body weight
Duncan et al. [26]	C57BL/6 J mice fed with HFD of 60% kcal fat	TRF	HFD ad libitum	Blood glucose, insulin, body weight
Bushman et al. [25]	C57BL/6 mice fed with HFD of 60% kcal fat	TRF (10 weeks)	HFD ad libitum	Blood glucose, lipid profile, body weight
Kim et al. [13]	C57BL/6 J mice fed with HFD of 60% kcal fat	TRF (6 weeks)	HFD ad libitum	Blood glucose, insulin, body weight
Lee et al. [23]	C57BL/6 J mice fed with HFD of 60% kcal fat	TRF (8 weeks)	HFD ad libitum	Blood glucose, insulin, body weight
Yun et al. [24]	C57BL/6 J mice fed with HFD of 60% kcal fat	TRF (6 weeks)	HFD ad libitum	Blood glucose, insulin, body weight
Kentish et al. [2]	C57BL/6 mice fed with HFD of 60% kcal fat	TRF (LP vs DP, 8 weeks)	HFD ad libitum	Body weight
Aouichat et al. [30]	Wistar rats fed with HFD of 47% kcal fat	TRF (16 weeks)	CAF ad libitum	Lipid profile, body weight
Azemi et al. [29]	Sprague–Dawley rats fed with HFD of 60% kcal fat	TRF (6 weeks)	HFD ad libitum	Blood glucose, insulin, lipid profile, body weight
Olsen et al. [28]	Sprague–Dawley rats fed with HFD of 60% kcal fat	TRF (12 weeks)	HFD ad libitum	Body weight
Bilibio et al. [31]	Wistar rats fed with HFD of 60% kcal fat	TRF (8 weeks)	HFD ad libitum	Blood glucose, lipid profile, body weight

### Search results

**Fig. 1:**
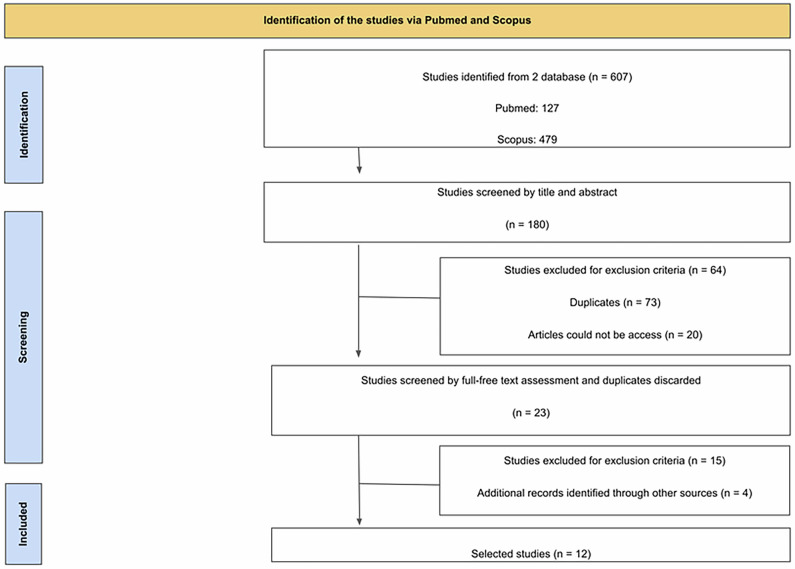
Illustrates the various phases of the systematic review and meta-analysis, detailing the number of records identified, screened, assessed for eligibility, and ultimately included in the final analysis.

The search results identified 607 publications using the descriptors. However, only 180 publications were selected after screening the title and abstracts. From the 180 publications, 73 papers were removed due to duplications, 64 papers were removed as they did not meet the criteria while 20 papers could not be fully accessed. Subsequently, only 23 publications were able to be fully accessed. From the 23 publications, 15 papers were rejected as some studies involved circadian clocks, other types of IF and no induction of obesity. An additional four papers were identified and included during the screening process by thoroughly reviewing the references of the screened journals. Hence, only 12 publications were available for the data collection after duplicates were removed (Fig. 1).

## Results

### Strain

In mice, two studies [22] and [2] utilized C57BL/6 while another six studies utilized C57BL/6 J mice as the model for DIO [13, 23, 24, 25–27]. From the four studies analyzed in rats, it was identified that Sprague-Dawley and Wistar are the 2 rat strains used [28–31].

### Fat content of HFD

From the eight articles, seven studies fed the mice with a HFD of 60–61% kcal of fat [13, 23, 24, 2, 25–27] while one study by [22] used a HFD of 42% of kcal of fat in mice. From the four studies in rats, only three studies used a HFD of 60% kcal of fat [28, 29, 31] while only one study by [30] used HFD of 47% kcal of fat (Table 4).

**Table 4:** Summary of various studies on DIO models, rodent strain, detailing the diet composition, duration and the findings obtained on blood glucose and insulin level, lipid test and body weights.

Pub.	Species/strain	Obesity induction		TRF treatment		Findings obtained		
**Author**	**Mice/Rat Strain**	**Diet (Duration)**	**Weight gain**	**Hours (feeding)**	**Weeks**	**Blood glucose and insulin level**	**Lipid profile level**	**Body weight**
Sherman et al. [22]	Mice C57BL/6	42% kcal from fat (2 weeks)	^a^Not reported	4	18 weeks	**TRF-HFD:** Normalized level of glucose, insulin resistance decreases **TRF-LFD**: Glucose decreases, insulin resistance decreases	**TRF-HFD:** TG no change, TC decreases, HDL decreases **TRF-LFD:** TG decreases, TC decreases, HDL decreases	**TRF-HFD:** 18% lower than the HFD *ad libitum* group **TRF-LFD:** Decreases
Hatori et al. [27]	Mice C57BL/6 J	61% kcal from fat (4 weeks)	^a^Not reported	8	18 weeks	**TRF-HFD:** Normalized blood glucose level, insulin level comparable to control group **TRF-NC:** No change	^a^Not reported	**TRF-HFD:** Body weight decreases **TRF-NC:** No change
M. Duncan et al. [26]	Mice C57BL/6 J	60% kcal from fat (^a^Not reported)	Not reported	8	^a^Not reported	**TRF-HFD:** Improved glucose tolerance, no effect on fasting insulin	^a^Not reported	**TRF-HFD:** Body weight decreases
Bushman et al. [25]	Mice C57BL/6	60% kcal from fat (18 weeks)	^a^Not reported	10	10 weeks	**TRF-HFD:** Glucose level decreases	**TRF-HFD:** TG increases	**TRF-HFD:** 4% lower than initial body weight
Kim et al. [13]	Mice C57BL/6 J	60% kcal from fat (6 weeks)	100%	10	6 weeks	**TRF-HFD:** Glucose decreases, insulin resistance decreases **TRF-HFD** + **L:** Glucose decreases, insulin resistance decreases	^a^Not reported	**TRF-HFD:** 17.1% lower than HFD-AL **TRF-HFD** + **L:** 18.5% lower than HFD-AL
Lee et al. [23]	Mice C57BL/6 J	60% kcal from fat (6 weeks)	100%	10	8 weeks	**TRF-HFD:** Glucose tolerance increases, insulin resistance decreases	^a^Not reported	**TRF-HFD:** 16.8% lower than the HFD group
Yun et al. [24]	Mice C57BL/6 J	60% kcal from fat (6 weeks)	100%	10	6 weeks	**TRF-HFD:** Glucose decreases, insulin resistance decreases	^a^Not reported	**TRF-HFD:** Body weight decreases
Kentish et al. [2]	Mice C57BL/6	60% kcal from fat (4 weeks)	41.2%	Light phase: 12 Dark phase: 12	8 weeks	NR	^a^Not reported	**TRF-DP:** Gained more weight than the HFD-LP mice.
Aouichat et al. [30]	Rats Albino Wistar	47% kcal from fat (Conducted concurrently with TRF)	^a^Not reported	8	16 weeks	NR	**TRF-CAF:** TG decreases 19.8%, TC decreases 27.8%, and LDL decreases 43.5% but HDL increases by 29.6%	**TRF-CAF:** Body weight decreases
Azemi et al. [29]	Rats Sprague - Dawley	60% kcal from fat (6 weeks)	37.8%	8	6 weeks	**TRF-HFD:** Glucose level decreases, no effect on insulin level	**TRF-HFD:** TG, TC, LDL decreases, no effect on HDL	**TRF-HFD:** Body weight decreases by 7.89%
Olsen et al. [28]	Rats Sprague – Dawley	60% kcal from fat (12 weeks)	300%	9	5 weekdays for 12 weeks	NR	^a^Not reported	**TRF-HFD:** Body weight decreases
Bilibio et al. [31]	Rats Wistar	60% kcal from fat (8 weeks)	35.71%	10	8 weeks	**TRF-HFD:** No change in glucose level	**TRF-HFD:** Lower serum cholesterol concentration, no changes in TG, LDL and HDL	**TRF-HFD:** Reduction of 0.93% in body weight change

*TRF-HFD* High-fat diet with TRF implementation, *TRF-LFD* Low-fat diet with TRF implementation, *TRF-NC* TRF with normal chow, *TRF-HFD* *+* *L* High-fat diet with TRF implementation and luteolin, *TRF-DP* Dark phase restricted high-fat diet, *TRF-LP* Light phase restricted high-fat diet, *TRF-CAF* Cafeteria diet with TRF implementation, *TG* Triglyceride, *TC* Total cholesterol, *HDL* High-density lipoprotein, *LDL* Low-density lipoprotein, *NR* Not reported.

^a^Indicates the parameter was not measured in the study.

### Duration of obesity induction (weeks)

The shortest duration used to induce obesity in mice is 2 weeks by [22] while the longest duration is 18 weeks by [25]. There are also studies that used different durations such as 4 weeks [2, 27] and 6 weeks [13, 23, 24]. On the other hand, in rats, the shortest duration taken to induce obesity in rats is 6 weeks [29] whilst the longest is 16 weeks [30]. There are also studies that induced obesity at 8 [31] and 12 weeks [28] in rats.

### Percentage of weight gain

From the eight studies of obesity induction in mice, four studies did not mention the percentage of weight gain upon HFD [22, 25–27]. The four studies with bodyweight gain data reported either 100% increase relative to baseline body weight [13, 23, 24] or 41.2% [2] weight gain after consumption of 60% kcal of HFD. The highest percentage of weight gain in mice was achieved after 6 weeks [13, 23, 24]whilst 41.2% of weight gain was achieved after 4 weeks [2].

In contrast, four studies in rats conducted the induction of obesity for a minimum of 6 weeks and maximum of 30 weeks. However, only three studies presented data on the percentage of weight gained. An induction of obesity carried out for 12 weeks demonstrated the highest weight gain of 300% [28]. The other two studies reported an increase of body weight of more than 35% after 6 [29] and 8 [31] weeks of obesity induction using HFD of 60% kcal.

### TRF (hours) duration

The feeding duration during TRF intervention in mice ranges from 4 to 12 h. The shortest duration of TRF intervention is 20 h fasting/4 h feeding [22] and the longest TRF duration is 12 h fasting/12 h feeding [2]. Nevertheless, two studies conducted TRF with 8 h of feeding/16 h fasting [26, 27] and the remaining four studies conducted on TRF with 10 h of feeding/14 h fasting in mice [13, 23, 24, 25].

In studies using rats as a model, the shortest feeding duration is 6 h/18 h fasting while the longest is 10 h/14 h fasting. Two studies conducted TRF intervention in rats by restricting feeding to 8 h/16 h fasting daily during the dark phase [29, 30]. The remaining two studies reported TRF interventions with feeding restrictions of 9 h/15 h fasting [28] and 10 h/14 h fasting [31].

### Effect of TRF on body weight

The highest weight loss exceeding 17% was reported in two studies. The first study conducted TRF intervention with 4 h of restricted feeding/20 h fasting for 18 weeks leading to 18% (*p* < 0.05) weight reduction compared to HFD group [22] while the other study conducted the TRF intervention with 10 h of restricted feeding/14 h fasting for 6 weeks leading to 17.1% (*p* < 0.05) weight reduction [13]. The lowest weight loss (4%) was observed when TRF intervention with 10 h of restricted feeding/14 h fasting for 10 weeks (*p* < 0.05) [31]. Additionally, a weight loss of less than 17% (*p* < 0.05) was observed when the TRF intervention with restricted feeding for 10 h/14 h fasting for a duration of 8 weeks (16.8% weight reduction) [23]. Interestingly, a study conducted TRF intervention with 12 h of restricted feeding/12 h fasting for 8 weeks at light phase and dark phase revealed that mice weighed more when fed at dark phase [2]. Three other studies did not provide data on the percentage of body weight reduction [24, 26, 27].

Similar results were observed in the rats whereby a body weight reduction was reported in all the studies. Highest body weight loss was observed in 8 h of TRF/16 h fasting conducted for 6 weeks (*p* < 0.05) [29]. A body weight reduction of 0.93% (*p* < 0.05) was recorded in a 10 h TRF/14 h fasting conducted for 8 weeks [31]. The other two studies did not provide specific data on the percentage of body weight reduction [28, 30].

### Effect of TRF on glucose and insulin levels

One study conducted in mice did not report any data on glucose and insulin level. Four studies that carried out the TRF intervention with 10 h restricted feeding/14 h fasting showed a decreased glucose level decreases [13, 23, 24, 25] and from the four studies only three studies reported reduced in the insulin resistance [13, 23, 24]. Interestingly, 8 h of TRF intervention/16 h fasting effectively normalized blood glucose and insulin levels, making them comparable to those of the control [27]. In a 4 h TRF intervention/20 h fasting, a normalized glucose level with reduction in insulin resistance was recorded [22]. Contradictorily, another study with 8 h TRF intervention/16 h fasting reported an improvement in the glucose level with no changes on fasting insulin [26].

From the four studies in rats, two studies conducted in rats did not report any data on glucose and insulin levels [28, 30]. A TRF intervention carried out for 6 weeks in rats revealed a reduction in glucose level but no change on insulin level [29]. Nevertheless, no significant change in blood glucose level was recorded in 10-h TRF/14 h fasting [31].

### Effect of TRF on lipid profile

From the eight studies, only two studies reported the data for lipid profile in mice [22, 25]. A decrease in total cholesterol (TC) and high-density lipoprotein (HDL) levels however no changes in the triglyceride (TG) level was reported in 4 h of restricted feeding duration/20 h fasting [22]. Meanwhile, an increase in TG was recorded in 10 h of feeding duration/14 h fasting [25].

From the four studies, only one study did not report the data on the lipid profile level in rats [28]. A decreased level of TG, TC and low-density lipoprotein (LDL) was reported in two studies with TRF intervention of restricted feeding for 8 h/16 h fasting [29, 30]. Surprisingly no changes were identified in the TG, LDL and HDL levels even though a reduction was observed in cholesterol concentration in 10 h of TRF intervention/14 h fasting [31].

## Discussion

The prevalence of obesity has reached alarming levels in society, highlighting the need for a deeper understanding of its underlying mechanisms, physiological changes, and effects on body weight. Animal models serve as valuable tools for advancing knowledge in this field. Obesity is commonly induced by feeding HFD in strains that are highly susceptible to obesity such as C57BL/6 in mice [32]. Outbred Wistar and Sprague-Dawley rats are frequently employed for DIO due to their variability in susceptibility and insulin resistance [33]. In addition, rats are bigger in size than mice, hence it facilitates the evaluation of various parameters [33].

In research involving metabolic diseases, the C57BL/6 mice are considered as one of the best strains to imitate the metabolic disease in humans that occurs due to obesity [19, 34]. This strain of mice develops obesity, hyperinsulinemia, and hyperglycemia when fed with HFD diet ad libitum [34]. The original strain of C57BL/6, known as B6 and C57BL/6 J (B6/J) is from the Jackson Laboratory (JAX) [22]. When comparing glucose intolerance, the C57BL/6 J mice are identified to be more glucose intolerant than C57BL/6 N and exhibit high tissue adiposity [19]. The two groups of strains derived from C57BL/6 are C57BL/6 J and C57BL/6 N [35]. The substrains of C57BL/6 J consists of C57BL/6 J, C57BL/6 JBomTac and C57BL/6JRj which are from different vendors and laboratories. Most of the studies utilized C57BL6/J compared to the other two substrains due to the rapid progression in gaining weight when fed with HFD [19]. Besides, C57BL/6J substrain is proven to delay the glucose clearance during glucose and insulin tolerance test suggesting insulin resistance when fed with HFD [19].

Both 45% and 60% fat diets are considered as high-fats for rodents as the standard rodent usually consists of 10% fats. Mice fed with either a 45% or 60% fat diet do gain weight however mice fed with a 60% kcal diet gains weight within a shorter period of time. This saves time and reduces cost required for housing and caging the animals [36]. Hence, most researchers prefer a 60% fat diet as it is more effective compared to a 45% fat diet in inducing obesity [37].

Highest percentage of weight gain of 100% in mice was obtained in 6 weeks of induction when compared to 4 weeks of induction gaining only 41.2%. The 3 studies conducted the induction of obesity for 6 weeks while only 1 study conducted the induction of obesity for 4 weeks [13, 23, 24]. Interestingly, the induction of obesity carried out for a longer period of time increased the percentage of weight gain in mice despite using the similar fat content of HFD. This revealed that extending the period of inducing obesity in mice to 6 weeks can successfully induce obesity in mice by increasing the percentage of weight gained to more than 20%.

The highest percentage of weight gain of 300% in rats was obtained in 12 weeks of obesity induction with HFD whilst the lowest percentage of weight gain (35.71%) was reported following 8 weeks of induction [28, 31]. However, 6 weeks of induction has a higher percentage of weight gained (37.8%) when compared to 8 weeks of induction despite using HFD of 60% kcal of fat [29]. Sprague-Dawley and Wistar are commonly used models of rats in studies related to obesity. However, the variability of responses to HFD that exist between these strains directly leads them to different weight gain [17].

In this review, it was identified that longer fasting duration leads to a loss in body weight. An increased fasting duration results in reduced energy consumption. The insulin signaling that decreases during fasting incorporated with the shift from glucose to fat metabolism reduces adiposity [38]. During fasting, a metabolic shift in the liver takes place when the glycogen level is depleted. This shift involves the use of lipid and cholesterol fusion and fat storage as energy fuel [39]. As the body weight reduces, the insulin resistance starts to improve which subsequently causes improvement in glucose uptake and depletion in circulating glucose levels. From the 13 studies that demonstrated a reduction in body weight, only four studies revealed a reduction in insulin resistance [22, 23, 24, 27]. This corresponds with other findings revealing that TRF reduces hyperinsulinemia in mice [40]. Meanwhile, another two studies demonstrated that the insulin level was not affected despite a loss in body weight [26, 29]. In contrast to the findings of M. Duncan and his colleagues (2016), the results of an 8 h TRF trial/16 h fasting after 8 weeks of dietary treatments in middle-aged mice in the TRF group showed an increase in insulin sensitivity [41].

The circadian system plays a pivotal role in coordinating metabolic homeostasis by regulating physiological processes such as sleep–wake rhythm, cardiovascular function, hormone secretion, and energy balance. At the molecular level, these rhythms are governed by transcriptional–translational feedback loops involving core clock genes such as CLOCK, BMAL1, PER, and CRY, which synchronize peripheral clocks in metabolic tissues [42]. Environmental cues, including food intake, light exposure, and physical activity, act as zeitgebers that entrain these molecular oscillators. Disruption of circadian regulation perturbs nutrient-sensing pathways, including AMPK, SIRT1, and REV-ERBα, leading to impaired glucose utilization, altered lipid metabolism, and increased risk of obesity [42].

Evidence from rodent studies indicates that TRF can restore metabolic disturbances in obesity by realigning disrupted circadian rhythms [43–46]. Remarkably, TRF also improves metabolic function in obesity-induced mice lacking core circadian clock genes [43], suggesting that its benefits extend beyond canonical clock regulation. While clock-deficient models exhibit arrhythmic feeding behavior, TRF reinstates temporal organization of food intake, thereby re-establishing rhythmic metabolic control [43]. In addition, TRF exerts epigenetic regulation of pancreatic β-cell function, reversing insulin resistance even under circadian disruption [47]. These findings highlight feeding time as a dominant zeitgeber capable of driving systemic metabolic reprogramming and compensating for circadian misalignment, positioning TRF as a promising chrono-nutritional intervention against obesity.

TRF improves metabolic homeostasis by reprogramming glucose, lipid, protein, and nutrient-sensing pathways. TRF enhances CRY expression and suppresses nocturnal pCREB activity which in turn downregulates the gluconeogenic genes pyruvate carboxylase (PCX) and glucose-6-phosphatase (G6pc) that mediate the rate-limiting step of gluconeogenesis. This rhythmic interplay between pCREB oscillation and CRY expression synergistically suppresses gluconeogenic transcription, favoring glycolysis, anabolic pathways, and reduced glutathione synthesis [48, 49]. In parallel, TRF attenuates fatty acid synthesis, elongation, and desaturation while promoting mitochondrial β-oxidation through REV-ERBα–mediated repression of the lipogenic gene fatty acid synthase (FASN) [50]. At the protein metabolism level, TRF restores ribosomal protein S6 phosphorylation, a marker of protein synthesis and a downstream target of

AMPK and insulin/Akt signaling in both liver and muscle, with enhanced activation during the dark/feeding phase. Beyond these effects, TRF remodels multiple nutrient-sensing pathways dysregulated in clock-deficient mice on a high-fat diet, including the TCA cycle, amino acid metabolism, aminoacyl-tRNA biosynthesis, glycerophospholipid metabolism, and unsaturated fatty acid biosynthesis [51]. Collectively, these findings highlight TRF as a potent regulator of metabolic flexibility through circadian and nutrient-sensing mechanisms.

The highest weight reduction of 18% when compared to the HFD ad libitum group was recorded in 4 h of TRF intervention/12 h fasting carried out for 18 weeks in mice [22]. Other than that, a weight reduction of 17% was reported in 10 h of TRF/14 h fasting after 6 weeks of the intervention [13, 23]. Hence, extending the TRF treatment period to 18 weeks may demonstrate a greater percentage of weight lost. Interestingly, TRF conducted for longer hours (10 h feeding) achieved almost similar results in body weight loss within a shorter duration compared to TRF conducted in shorter hours (4 h feeding). A 10-h TRF/ 14 h fasting over 6 weeks on a 60% HFD resulted in a 17% reduction in body weight [13]. However, applying the same TRF protocol for 10 weeks produced only a 4% reduction [25]. Interestingly, extending the TRF duration did not increase the weight reduction. A 4 h TRF/ 14 h fasting for 18-week with a 42% HFD achieved an 18% decrease in body weight [22]. This emphasizes that caloric restriction is essential to achieve significant weight loss after 6 weeks. Caloric restriction reduces metabolic rate and energy expenditure (EE) resulted from reduced energy intake [52] Since EE is proportional to body weight, any weight loss further diminishes EE ultimately creating a new energy balance at a lower body weight [52]. This process is known as metabolic adaptation which aims in conserving energy. These adaptations include reductions in basal metabolic rate, energy expenditure, and alterations in substrate utilization. Consequently, this protects against excessive energy deficit but also reduces weight loss over time [52].

To achieve better results in weight reduction in a short duration, the protocol established by [23] is the most effective as the highest weight loss was reported in their study. In rats, the 8 h of TRF/16 h of fasting lead to the highest weight reduction of 7.89%. Meanwhile, a weight loss of 0.93% was recorded for 10 h of TRF/14 h fasting conducted for 8 weeks. Hence, adhering to the protocol by [29] is effective to achieve better results in weight reduction. Mice exhibit greater bodyweight reduction during fasting compared to rats primarily due to species-specific metabolic and physiological differences. Mice have a higher mass-specific metabolic rate and smaller energy reserves which results in increased susceptibility to rapid weight loss when nutrient intake is withheld. Their elevated locomotor activity and enhanced activation of brown adipose tissue thermogenesis further increase energy expenditure [32]. Conversely, rats possess larger adipose stores and a lower relative energy demand leading to greater resistance to fasting-induced weight loss [53].

Higher weight loss was identified in the mice compared to the rats [30]. This could be due to the fact that mice have high metabolic rates such as higher mass-specific oxygen consumption and carbon dioxide production which are indicators of metabolic rate under hypoxia [30]. Hence, mice can easily burn more energy at a higher rate per unit body mass than rats [30].

HFD triggers hyperlipidemia which causes the serum level of TC, TG and LDL to increase while TRF decreases the TG, TC and LDL [29]. A similar result was observed in rats in an 8 h TRF intervention/16 h fasting whereby a reduction in TG, TC, LDL with an increase in HDL was recorded [30]. This finding corresponds with other findings revealing that TRF reverses hyperlipidemia in mice fed with HFD [54]. Only one study recorded a decrease in TC and HDL most probably due to shorter TRF intervention [24]. Contradictorily, a 10-h TRF intervention/14 h fasting only reduced the levels of TC with no effect on TG, HDL and LDL in Wistar rats [31]. This may occur as TRF is not able to mitigate the metabolic deficits brought on by HFD consumption in middle-aged female rats with obesity [31]. Similar result was reported in another finding by [41] that revealed no changes in lipid profile level in middle-aged mice with obesity. Furthermore, a 10-h feeding duration/14 h fasting conducted using mice causes a rise in TG [25]. For LDL level, about 3%-5% of reduction can be observed when the body weight reduction is 5% while 20% reduction in TGs can be observed with 5–10% of body weight reduction [55].

Improved blood glucose levels are favorably correlated with changes in lipid level [56]. Out of 14 studies being reviewed, eight studies reported an improvement in the glucose tolerance with only one study revealed no changes on glucose level [13, 23, 24, 29, 22, 25–27]. A 10-h TRF regimen/14 h fasting improved blood glucose suggesting that TRF can positively impact glucose regulation [56]. Glycogenolysis is initiated during fasting as the pancreas secretes glucagon. This causes the glycogen which is stored as glucose to be released as glucose [57]. Long fasting duration causes the glycogen that is being stored in the liver to be reduced, creating a shift from metabolic lipid/cholesterol synthesis and fat storage to utilizing the fat via fatty acid oxidation [58]. In a 10-h feeding duration/14 h fasting using rats, a body weight reduction of less than 1% was observed with no significant changes in glucose levels [31]. This corresponds with other findings revealing that TRF conducted in older rats neither have effect on plasma glucose and insulin levels nor reverse the insulin resistance due to HFD [59].

Improvement in glucoregulatory level in mice was observed in five studies [13, 23, 24, 22, 27]. In 4 h/20 h fasting and 8 h of TRF intervention/16 h fasting carried out for 18 weeks, a reduction in glucose and insulin resistance was revealed [22, 27]. Similar results were also observed in 10 h of TRF intervention/14 h fasting carried out in a shorter period of time which is 6–8 weeks [13, 23, 24].

This demonstrates that shorter feeding duration 4–8 h/16–20 h fasting requires a longer period of TRF intervention while longer feeding duration 10 h/14 h fasting requires a shorter period of TRF intervention to achieve significant effects. Given that the study by [23] reported weight reduction and optimal results in glucoregulatory levels, it is recommended to adhere to their protocol for better results in glucose and insulin levels. In rats, only one study that carried out the TRF intervention (feeding) of 8 h/16 h fasting for 6 weeks revealed a reduction in glucose level [29] suggesting that this protocol can provide better results in glucoregulatory level.

In summary, obesity can be induced for 4–6 weeks in C57BL/6 mice using HFD of 60% kcal of fat to obtain a 41.2–100% weight gain. A six-week obesity induction period was required using 60% kcal HFD in C57BL/6 mice to obtain 100% weight gain [23].

The effect of TRF on weight loss can be observed in mice as early as 8 weeks into the intervention (10 h of feeding/14 h of fasting) leading to a 16.8% reduction in body weight [23]. Besides, improvement in glucoregulatory effect whereby increase in glucose tolerance and decrease in insulin sensitivity was reported. On the contrary, the highest weight loss (18%) was reported when TRF (4 h feeding/20 h fasting) duration is for 18 weeks [22]. Interestingly, no data was reported on the lipid profile levels in mice treated with TRF. Only one study has reported an improvement in glucoregulatory function which is indicated by normalized glucose levels and reduced insulin sensitivity, when the fasting duration is 20 h/4 h of feeding over a period of 18 weeks [22]. It can be observed that shorter fasting hours (14 h vs 20 h) obtained almost similar results in body weight reduction within 8 weeks.

On the other hand, obesity can be induced for 6–12 weeks in rats using HFD of 60% kcal of fat to obtain 35.71–300% of weight gain. Sprague-Dawley rats treated with HFD containing 60% kcal of fat for six weeks exhibited a weight gain exceeding 35% [31]. The highest weight gain (300%) was reported in Sprague-Dawley rats subjected to HFD for 12 weeks [28].

**Fig. 2:**
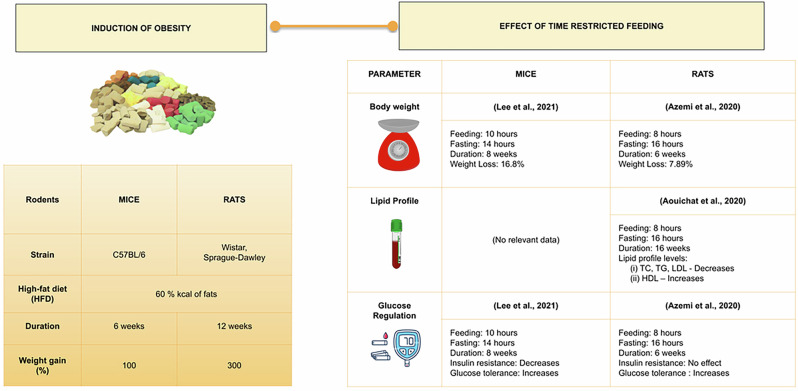
Obesity was induced in mice (C57BL/6; LFD *n* = 7, HFD-ad lib *n* = 7, HFD-TRF *n* = 7; Lee et al., 2021) and rats (Wistar, *n* = 6/group; Sprague-Dawley, *n* = 7/group; Aouichat et al. 2020; Azemi et al. 2022) by feeding a (HFD, 60% kcal from fats) for 6 weeks in mice and 12 weeks in rats leading to 100% and 300% weight gain respectively. Following obesity induction rodents underwent TRF which influenced body weight, lipid profile, and glucose regulation differently. In mice an 8-week TRF (10 h feeding/14 h fasting) resulted in 16.8% weight loss (*p < 0.05) and improved glucose tolerance (**p < 0.01). In Sprague-Dawley rats a 6 h feeding/18 h fasting regimen led to 7.89% weight loss (*p < 0.05). In Wistar rats TRF improved lipid profiles by reducing TC, TG, and LDL and increasing HDL (*p < 0.05). For glucose regulation TRF in mice reduced insulin resistance and improved glucose tolerance whereas in rats it had no effect on insulin resistance but improved glucose tolerance (*p < 0.05).

The effect of TRF intervention on weight loss can be observed as early as 6 weeks (8 h of feeding/16 h fasting) that leads to the highest weight loss of 7.89% [29]. Besides, the TG, TC and LDL in the lipid profile level were reduced as well. In terms of glucoregulatory level, the 8 h of TRF/16 h fasting increased the glucose tolerance. Meanwhile, the 16 weeks of TRF intervention with 8 h of feeding/16 h fasting reported a weight reduction as well [30]. Interestingly, the HDL levels were increased while the TG, TC and LDL levels were decreased. No data on the glucoregulatory levels were reported in this study. Interestingly, TRF intervention carried out for 8 weeks increased the HDL level compared to 6 weeks that significantly did not affect the HDL level. In conclusion, TRF significantly reduces body weight, increases glucose tolerance, reduces insulin sensitivity and improves lipid profile levels (TG, TC, HDL and LDL) (refer Fig. 2).

Currently, several drugs are approved for long-term treatment of obesity worldwide which include Orlistat (Xenical, Xenical, Roche, Basel, Switzerland), 1999; Bupropion/Naltrexone (Contrave, Orexigen Therapeutics Inc., La Jolla, CA, USA), 2018; Liraglutide 3.0 mg (Saxenda, Novo Nordisk, Bagsvaerd, Denmark), 2018; Phentermine/Topiramate (Qsymia,Vivus, Mountain View, CA, USA), 2019; Semaglutide 2.4 mg (Wegovy, Novo Nordisk), 2021; Tirzepatide (Zepbound, Eli Lilly, Indianapolis, IN, USA), 2023 [60]. Among incretin-based therapies, GLP-1 receptor agonists (GLP-1RAs) and dual GLP-1/GIP co-agonists have attracted considerable attention due to their robust weight-lowering efficacy. GLP-1, secreted by intestinal L-cells in response to nutrient intake acts on receptors expressed in the pancreas, liver, muscle, and brain. It enhances glucose-dependent insulin secretion from β-cells, suppresses glucagon release from α-cells, delays gastric emptying, and promotes satiety which collectively contributes to significant weight reduction [61].

Lifestyle intervention regime such as TRF offers a safe, non-invasive, and cost-effective approach that improves weight and metabolic outcomes, but its effects are modest compared to bariatric surgery. Bariatric surgery remains the most effective intervention for severe obesity, achieving greater and more durable weight loss, higher remission rates of type 2 diabetes and metabolic syndrome, and reduced medication use, albeit with risks such as nutritional deficiencies and reoperations [62]. These strategies may be considered complementary, with TRF suitable for early or moderate obesity management and bariatric surgery reserved for more severe or refractory cases.

## Conclusion

In this study, C57BL/6 strains in mice, Sprague-Dawley and Wistar rats are used in research to explore the effects of TRF in obesity in vivo models. Obesity was induced by feeding the mice and rats with 60% of HFD for 4–6 weeks in mice and 6–12 weeks in rats to increase the caloric intake. As the caloric intake increases, subsequently the body weight increases leading to obesity within a short period of time. The increase in body weight directly impacts the body metabolism which are the lipid profile levels and the glucoregulatory levels. Furthermore, 10 h of TRF/14 h fasting for 8 weeks in mice improves the metabolic parameter as depicted by reduced body weight and glucose level and improved insulin and lipid profile levels. However, the findings are limited, as some reviewed studies did not provide adequate evidence regarding glucoregulation, body weight, and lipid profiles.

TRF appears as an alternative technique compared to other diet restrictions and pharmacological therapy that may present risks. TRF may appear a several tissues or organs. The lack of sex-stratified data with most studies using male animals limits the generalizability of the findings. It is also noteworthy to highlight the presence of publication bias that may have influenced the available evidence on TRF. Studies reporting positive outcomes are more likely to be published than those with null or negative findings. This eventually could lead to an overestimation of the benefits of TRF. cost-effective and universally applicable treatment to manage obesity which can contribute to the development of a healthier nation. Future research on obesity may also take into account the inclusion of different metabolic challenges in order to assess whether the advantages of TRF are exclusive to any one of the challenges or multiple challenges that contribute to obesity.

## Limitations

The interpretation of the current evidence on TRF is constrained by several limitations that warrant careful consideration. To date, all studies reported a significant reduction in body weight and glucose levels. However, the absence of consistent data on insulin sensitivity and lipid regulation prevents conclusive interpretations regarding the effects of TRF on metabolic health. Obesity is well reported to alter glucose, insulin, and lipid metabolism and the lack of data on these parameters further limits a comprehensive understanding of TRF effects in obesity. This gap highlights the need for future studies to incorporate standardized measurements of insulin and lipid parameters to better elucidate the mechanisms underlying TRF-induced metabolic improvements. Secondly, fewer studies were conducted using rats which is only four while eight studies were conducted using mice. The choice of selection among these two species relies on size and cost as mice are smaller and cheaper compared to rats. In particular, C57BL/6 mice are known to be prone to obesity and are preferred to use in research to identify how obesity impact several tissues or organs. The lack of sex-stratified data with most studies using male animals limits the generalizability of the findings. It is also noteworthy to highlight the presence of publication bias that may have influenced the available evidence on TRF. Studies reporting positive outcomes are more likely to be published than those with null or negative findings. This eventually could lead to an overestimation of the benefits of TRF.
